# Disparities in Drinking Water Manganese Concentrations in Domestic Wells and Community Water Systems in the Central Valley, CA, USA

**DOI:** 10.1021/acs.est.2c08548

**Published:** 2023-01-25

**Authors:** Miranda L. Aiken, Clare E. Pace, Maithili Ramachandran, Kurt A. Schwabe, Hoori Ajami, Bruce G. Link, Samantha C. Ying

**Affiliations:** † Environmental Toxicology Graduate Program, University of California, Riverside, California 92521, United States; ‡ Environmental Science, Policy, and Management, University of California, Berkeley, California 94704, United States; § School of Public Policy, University of California, Riverside, California 92521, United States; ∥ Environmental Sciences Department, University of California, Riverside, California 92521, United States; △ Schmid College of Science and Technology, Chapman University, Orange, CA 92866, United States; ▽ Health Disparities Research Center, University of California, Riverside, California 92521, United States

**Keywords:** human right to water, secondary data, well depth, redox conditions, community water systems, domestic well communities

## Abstract

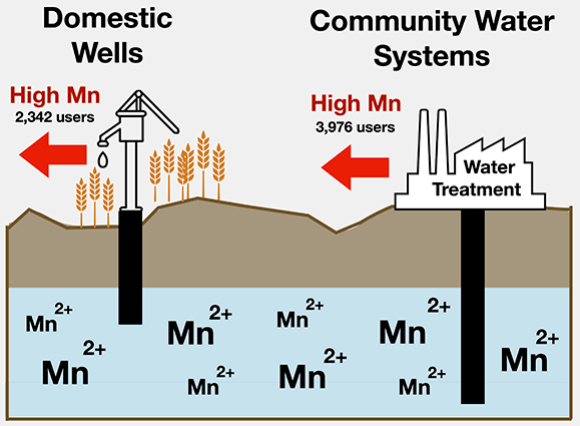

Over 1.3 million Californians rely on unmonitored domestic wells. Existing probability estimates of groundwater Mn concentrations, population estimates, and sociodemographic data were integrated with spatial data delineating domestic well communities (DWCs) to predict the probability of high Mn concentrations in extracted groundwater within DWCs in California’s Central Valley. Additional Mn concentration data of water delivered by community water systems (CWSs) were used to estimate Mn in public water supply. We estimate that 0.4% of the DWC population (2342 users) rely on groundwater with predicted Mn > 300 μg L^–1^. In CWSs, 2.4% of the population (904 users) served by small CWSs and 0.4% of the population (3072 users) served by medium CWS relied on drinking water with mean point-of-entry Mn concentration >300 μg L^–1^. Small CWSs were less likely to report Mn concentrations relative to large CWSs, yet a higher percentage of small CWSs exceed regulatory standards relative to larger systems. Modeled calculations do not reveal differences in estimated Mn concentration between groundwater from current regional domestic well depth and 33 m deeper. These analyses demonstrate the need for additional well-monitoring programs that evaluate Mn and increased access to point-of-use treatment for domestic well users disproportionately burdened by associated costs of water treatment.

## Abstract 2

Thousands of domestic well and community water system users in California’s Central Valley are likely accessing manganese-contaminated groundwater without the economic means to treat it.

## 1 Introduction

Manganese (Mn) is a ubiquitous groundwater constituent resulting from the solubilization of naturally occurring mineral sources.^1,2^ While manganese concentrations in the range of 0.4–550 μg L^–1^ are common in untreated groundwater, levels as high as 28,200 μg L^–1^ have been reported in the United States.^2,3^ Currently, Mn has a federal secondary maximum contaminant level (SMCL) of 50 μg L^–1^,^4^ a federal health advisory limit (HAL) of 300 μg L^–1^,^5^ and in California, a customer notification level of 500 μg L^–1^ for community water systems.^6^ In 2021, the World Health Organization reissued a provisional guideline of 80 μg L^–1^ for Mn in drinking water after removing the previous guideline (400 μg L^–1^) in 2012. This revision was due to the further accumulation of health studies linking Mn concentrations in drinking water with negative health impacts and was set to be protective of bottle-fed infants, the most vulnerable population.^7^

A growing body of evidence suggests that Mn concentrations previously considered safe may pose significant health threats to vulnerable populations such as children. Studies of school-aged children consuming drinking water with naturally elevated concentrations of Mn have demonstrated lower academic achievement scores when their drinking water Mn concentrations exceeded 400 μg L^–1^,^8^ as well as poor memory, attention, and increased risk of attention deficit hyperactivity disorder when Mn concentrations exceeded 100 μg L^–1^ in drinking water.^9−11^ Despite these studies, the US EPA maintains a secondary contaminant status for Mn on the strength of adult cohort studies that did not observe neurotoxic endpoints.^5^ In 2020, Mn was sampled in a subset of small and large public water systems under the Unregulated Contaminant Monitoring Rule (UCMR4). Approximately 2.1% of the water systems sampled reported Mn concentrations greater than the federal HAL.^12^

Communities served by domestic wells rely on unregulated groundwater and face potentially greater risks of contaminant exposure than community water system users due to the relative lack of regulatory oversight of these systems by state and federal drinking water agencies.^13^ Domestic wells are generally drilled to a shallower depth than public wells and therefore have redox conditions that favor Mn dissolution.^2,14,15^ Shallow aquifers are more susceptible to drought leading to well failure and an increased concentration of redox-sensitive contaminants as groundwater levels continue to decline.^16,17^ Several recent studies demonstrate that a disproportionate number of communities reliant on domestic wells in California are disadvantaged communities that likely face financial challenges in testing and treating groundwater.^13,18^ Furthermore, Mn concentrations above 500 μg L^–1^ were reported in untreated groundwater in 47 out of 58 counties in California between 2011 and 2019.^6^ For these reasons, private wells drilled within shallow aquifers deserve further consideration when assessing groundwater quality and mitigation efforts.

The focus of this research is on California’s Central Valley, a region recognized as one of the most productive and economically important agricultural regions in the United States and currently home to one-third of domestic well users in the state.^16^ Most groundwater basins in the Central Valley are defined as “critically overdrafted” by the California Sustainable Groundwater Management Act (SGMA) and are currently undergoing sustainability planning to address continued overdraft while meeting strict water quality standards for users.^19,20^ A projected population growth of 3.5 million within California by 2030, coupled with an agricultural sector confronting lower surface water supplies due to climate change, will exert increasing demand on California’s already stressed groundwater systems.^21^

The intention of this study is to characterize the communities at risk of exposure to unsafe levels of Mn contamination in their groundwater-sourced drinking water and possible barriers to exposure mitigation. Specifically, our study will (1) characterize Mn concentrations in delivered community water system drinking water, (2) predict the probability of high Mn concentrations in extracted groundwater within domestic well communities, (3) evaluate sociodemographic characteristics in populations served by domestic wells with a high likelihood of Mn above health-based thresholds, and (4) investigate the availability and effectiveness of mitigation measures such as increased well depth, point-of-use treatment, and consolidation of water systems for domestic well users relying on groundwater with Mn exceeding threshold values.

## 2 Materials and Methods

To best characterize the domestic well communities (DWC) and community water systems (CWS) accessing groundwater with high Mn concentrations, we integrated water source type boundaries (i.e., CWS or DWC), with population estimates served by each water source, poverty estimates, and water quality predictions. Since CWSs treat water before delivery, reported water quality data at point of entry (last point where water quality was measured prior to entering the delivery system) was integrated with available spatial delineations of water-use-type boundaries. A summary of the spatial resolution of available data is listed in Table 1, and links to all publicly available data sets are listed in Table S1.

**Table 1:** Summary of Spatial Integration of Available Data

purpose	description	initial spatial resolution	integration	final spatial resolution
water system	likely DWC boundaries^a^	PLSS Grid	PLSS grids outside of CWS boundaries, populated, and containing a reported well were designated as a DWC	DWC boundary
	CWS boundary^a^	CWS boundary	active systems designated as community water systems were retained	CWS boundary
water quality	groundwater Mn predictive model (33 m, 50 m, 67 m, 84 m, 100 m)^b^	1 km^2^ Raster	mean predicted probability of groundwater Mn exceeding threshold values within CWS or DWC polygon boundaries calculated at depth	DWC or CWS boundary
	reported Mn concentrations at point most proximal to point of entry	CWS boundary	reported Mn concentrations matched with boundary by CWS ID	CWS boundary
population	US Census population	Census Block	aerial apportionment of 2010 US Census population^a^	DWC boundary
	CWS user population	CWS Boundary	reported values from SDWIS	CWS boundary
socioeconomics	percentage below 2FPL (OEHHA)	Census tracts	aerial weighted apportionment of reported values	DWC boundary
	disadvantaged community designation	County	retained designation if >50% of DWC boundary was within DAC community	DWC boundary

a From Pace et al.^25^

b From Rosecrans et al.^27^

### 2.1 Community Water Systems

A community water system is defined as a system providing water for human consumption with 15 or more service connections or serving 25 or more people daily for at least 60 days per year (HSC § 116275). CWS boundaries were obtained from the Drinking Water Tool in October 2019.^22^ In brief, boundaries from Tracking California Water System Service Area Tool were cleaned by removing duplicates, resolving overlaps, selecting active systems,^23^ and excluding wholesalers^24^ because they do not distribute directly to consumers. The final geospatial layer contained 2,851 active CWSs within California and 667 within the Central Valley alluvial aquifer boundary.^25^ CWSs were stratified by the number of service connections into small (15–199 connections), medium (200–9999 connections), and large (10,000+ connections) water systems.^13,26^

### 2.2 Domestic Well Communities

Domestic well communities are defined as populated Public Land System Survey (PLSS) grids that are (1) within populated portions of a Census block, (2) outside the boundaries of community water system service areas, (3) intersect with at least one domestic well according to the Department of Water Resources Online System for Well Completion Reports (OSWCR) database, and (4) intersect with at least one residential parcel.^25^ Some limitations in the dataset include the absence of small water systems due to a lack of publicly available data, missing/misclassified wells in the OSWCR database, and limitations in aerial apportionment of census data from census blocks to PLSS section geography in rural areas with large census blocks and low population. DWCs data were retrieved in March 2020.

### 2.3 Groundwater Mn Concentration Prediction Model

Due to the lack of reported water quality values for domestic wells within California’s Central Valley (Table S5), we used predicted probability grids of groundwater Mn concentrations at multiple depths and 1 km^2^ resolution developed for this region to estimate the likelihood that untreated groundwater exceeded threshold concentration of Mn.^27^ In brief, a Boosted Regression Tree (BRT) method^28^ generated probability of groundwater Mn concentration using over 60 subsurface geochemical and hydrological variables (e.g., regional soil properties, soil chemistry, land use, aquifer textures, and aquifer hydrology) within the Central Valley alluvial aquifer boundary. The resulting raster grids estimate the probability of Mn concentration exceeding 50 μg L^–1^ (SMCL) or 300 μg L^–1^ (HAL) at multiple depths. Here, we compared Mn exceedance probability estimates at depths corresponding to the median value of domestic wells within each hydrologic region^29,30^ in the Central Valley (Sacramento River, 33 m; San Joaquin River, 50 m; Tulare Lake, 66 m; Table S4) against predictions associated with well depth during drought conditions (17 m deeper than current median DWC well depth) and deep wells (33 m deeper than current median DWC well depth). Additional discussion of the BRT model methods, output, other assumptions, and limitations are provided in Rosecrans et al.^27^

### 2.4 Water Quality Estimation in Community Water Systems

Water quality for CWSs was estimated using reported data collected from the Safe Drinking Water Information System (SDWIS) over the most recent regulatory cycle of 2011–2019.^23^ All active facilities designated as community water systems were retained for analysis.^23^ Values reported as lower than the reported laboratory limit of detection (68.5% of total reported values) were calculated to be the reporting limit divided by the square root of 2.^26,31^

To account for water blending prior to distribution and best estimate contaminant concentration at point of use, only reported values that flowed directly into distribution systems (e.g., after blending and treatment) were retained and herein referred to as the most proximinal to point of entry. Flow path data from the Division of Drinking Water were used to identify the location of reported values within the flow path from the extraction of raw water, treatment, and point of entry. Any data sampled from a source that did not flow directly into the distribution system or did not have reported flow path data were excluded from further analysis.^32,33^ To account for higher-frequency sampling when in exceedance, samples collected on the same day and sampling location were averaged. A 9-year time-weighted average was calculated for each water system to allow comparison since reporting frequency was highly heterogeneous between systems.

### 2.5 Population Estimates and Sociodemographics

Population data from SDWIS Public Water System Information was used to estimate population exposure to concentrations above threshold values.^23^ Only residential users were included in population calculations. Any CWS with fewer than 15 service connections were assumed to be a state-classified small system and excluded from the analysis. The population within CWS service area boundaries were assumed to access public water exclusively and do not rely on private wells. To estimate populations reliant on CWS with mean Mn concentrations exceeding threshold values, the reported user population within these systems was summed. Population within domestic well communities was estimated via aerial apportionment of the 2010 United State Census block population by Pace et al.^25^ and retained for our analysis.

We estimated poverty using 2011–2015 American Community Survey (ACS) data and methods developed by the Office of Environmental Health and Hazard Assessment for CalEnviroScreen 4.0.^34^ We estimated the percentage of individuals falling below 200% of the Federal Poverty Level (2FPL). This translates to an income of $25,760 for an individual and $53,000 for a family of four. Individuals or families with incomes less than twice the FPL are eligible for many social assistance programs such as Medicaid. Census tract boundaries were overlaid with the DWC delineations to compare poverty rates between DWCs within and outside of the Central Valley. If DWC delineations overlapped with multiple census tract boundaries, a poverty value was assigned based onaerial apportionment. Hydrologic region boundaries were from CalWater.^35^

We assigned disadvantaged community (DAC) status at the county level using the 2017 designation by CalEPA.^36^ DACs face the top 25th percentile environmental, socioeconomic, and health burdens throughout the state and are eligible for access to additional state funding to address these disparities.

### 2.6 Statistical Analysis

We compared the probability of Mn exceeding the SMCL and HAL within DWCs at the current average depth of wells in each hydrologic region (Sacramento River, 33 m; San Joaquin River, 50 m; Tulare Lake, 66 m) versus drought conditions (17 m deeper than current) or deeper well depths (33 m deeper) using a set of paired *t*-tests. To test for significant differences in predicted Mn concentration in groundwater between Central Valley DWC users within or outside of disadvantaged communities, we used a Welch *t*-test. We also examined the hypothesis that poverty rates (i.e., the share of population living below 2FPL) in DWCs within the Central Valley are higher than poverty rates in DWCs elsewhere in the state of California. An α value of 0.05 is used for each test and all analyses were performed in RStudio (v 2022.02.1).

## 3 Results and Discussion

### 3.1 Measured Water Quality in Community Water Systems

**Figure 1:**
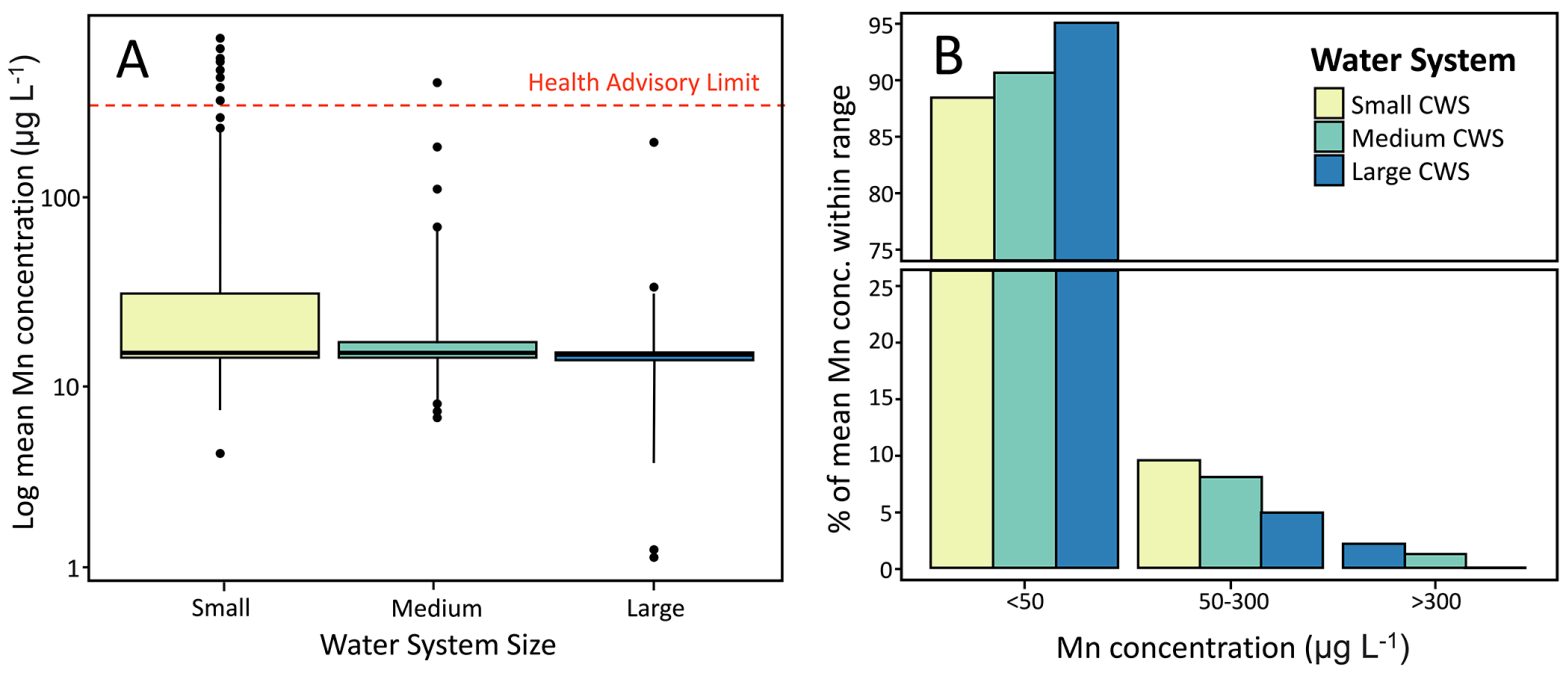
(A) Distribution of mean Mn concentrations at the point most proximal to point of entry between 2011 and 2019 for small, medium, and large CWSs using data from EPA Safe Drinking Water Information System (SDWIS). Boxes represent the 25th, 50th, and 75th percentiles of concentrations, and whiskers represent 5th and 95th percentiles. Outliers are represented by points. The red dashed line is the health advisory limit (300 μg L^–1^). Note that mean Mn concentrations are provided on log scale. (B) Percentage of mean Mn concentration between 2011 and 2019 that were below or in exceedance of threshold values (SMCL of 50 μg L^–1^ and HAL of 300 μg L^–1^). CWS = community water system. Small CWS = 15–199 service connections (*n* = 421); medium CWS = 200–9999 service connections (*n* = 193); large CWS ≥ 10,000 service connections (*n* = 53). Data from Safe SDWIS.

Our results show that smaller water systems reported Mn concentration less frequently than larger systems and have a higher percentage of total population relying on water with Mn concentration greater than the health advisory limit. An estimated 8.7 million residential customers in the Central Valley rely on water delivered by the 667 CWS. Of these systems, 421, 193, and 53 are categorized as small, medium, and large, respectively. However, only 373—approximately 55%—of CWS systems reported Mn concentrations between 2011 and 2019 despite regulation of secondary contaminants requiring reporting every 1–3 years (Table 2, 22 CCR § 64449). Previous studies demonstrate less frequent reporting of primary contaminants by water system size, with smaller systems reporting less frequently than larger systems.^26,37,38^ In the present study, we similarly found large water systems reported Mn concentration at the point most proximal point of entry 18.9 times per year compared to just 1.1 annual reports by small water systems. Approximately 58.2% of small CWS and 45.1% of medium CWS reported one or more Mn measurements at the point most proximal to point of entry over the entire 9-year study period. Data availability was slightly better for large water systems, with 77.4% of large water systems reporting at least one point of entry Mn measurement between 2011 and 2019. Across all CWS sizes, the mean Mn concentration exceeded the secondary notification limit of 50 μg L^–1^ for approximately 80,184 users and exceeded the health limit of 300 μg L^–1^ for 3976 users. However, this is likely an underestimate due to the lack of available data. Among the system sizes, 51 small systems, 8 medium systems, and 1 large system had a mean reported Mn concentration into the distribution system exceeding 50 μg L^–1^. Although a larger number of users accessing water exceeding the SMCL and HAL are in medium and large systems, a larger percentage of smaller CWS were distributing water exceeding regulatory standards (Figure 1).

**Table 2:** Mn Concentration Data in Groundwater Serving DWCs and Delivered by CWS and Predicted Population Exposed to Concentrations Exceeding Threshold Values between 2011 and 2019

	total DWC^a^	Sacramento River DWC	San Joaquin River DWC	Tulare Lake DWC	small CWS^b^	medium CWS^b^	large CWS^b^
service connections^c^	1–4				15–200	200–9999	10,000+
total CWS or wells in Central Valley	69,733 wells				421 CWS	193 CWS	53 CWS
total CWS with reported point of entry Mn values (% of total)^d^					245 (58.2) CWS	87 (45.1) CWS	41 (77.4) CWS
count of reported Mn values most proximal topoint of entry^d^					2,323	4,736	6,054
observations per CWS per year					1.1	6.9	18.9
total population with reported values					38,424	848,497	6,116,365
population (%) <50 μg/L					31,558 (82.1)	810,809 (95.5)	6,080,735 (99.4)
population (%) 50–300 μg/L					5962 (15.5)	34,616 (4.1)	35,630 (0.6)
population (%) >300 μg/L					904 (2.4)	3,072 (0.4)	0 (0)
total population with predictive model values^e^	549,718	156,567 (28.5)	234,807 (42.7)	158,344 (28.8)			
population (%) >80% probability of exceeding 50 μg/L Mn	22,468 (4.1)	17,872 (11.4)	3538 (0.2)	1058 (0.7)			
population (%) <80% probability of exceeding 50 μg/L Mn^e^	527,250 (95.9)	138,695 (88.6)	231,269 (99.8)	157,286 (99.3)			
population (%) >80% probability of exceeding 300 μg/L Mn^e^	2259 (0.4)	189 (0.1)	2011 (0.1)	59 (0.04)			
population (%) <80% probability of exceeding 300 μg/L Mn^e^	547,459 (99.6)	156,378 (99.9)	232,796 (99.9)	158,285 (99.96)			

a DWC = Domestic Well Communities.

b CWS = Community Water Systems.

c Size cutoffs are from Pace et al.^13^ and Bangia et al.^59^

d Total reported Mn values state-wide and most proximal to point of entry are available in Table S2.

e Population values from Pace et al.^25^

### 3.2 Predicted Water Quality in Domestic Wells

Due to a paucity of data on reported Mn concentrations in supplying domestic wells (Table S5), we do not report groundwater Mn concentration for DWCs in Table 2, and instead, report population with a probability above or below 80% likelihood of withdrawing groundwater in exceedance of 50 μg L^–1^ Mn or 300 μg L^–1^Mn. Rosecrans et al.^27^ predictions correspond with 93.4% of the area served by domestic wells in the Central Valley and 90.1% of the domestic well population within this region. We estimate that 549,718 individuals in the Central Valley alluvial aquifer boundary region rely on domestic wells (Table 2). Additional analyses using population estimates provided in Johnson et al.^39^ were also assigned to DWCs. These population estimation methods predicted fewer users within the Central Valley region (311,981 users); however, a similar percentage of users accessing groundwater with a high probability of Mn exceeding 300 μg L^–1^ (Table S12). We hypothesize the discrepancy in the population estimates derives from the differences in methods. Pace et al.^25^ used aerial apportionment of the population from the 2010 U.S. Census in geographical locations without access to CWS, whereas Johnson et al.^39^ extrapolated the percentage of users within census blocks relying on private wells from the 1990 U.S. Census to 2010 U.S. Census. Estimates derived from Pace et al.^25^ were within the range of other domestic well user estimates at the county level within the region^13,39^ (452,450–686,000 users).

**Figure 2:**
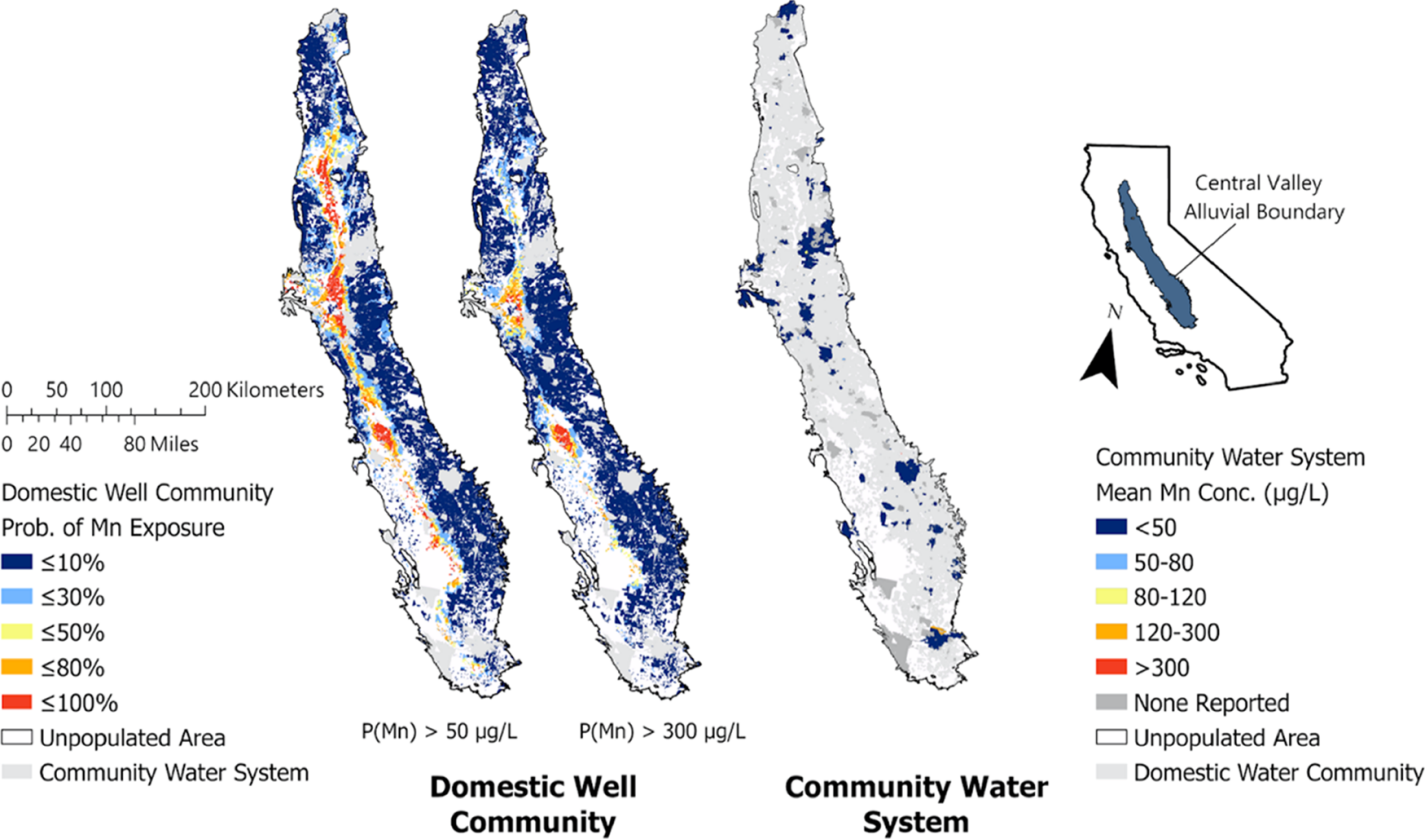
Probability map of groundwater Mn > 50 μg L^–1^ (SMCL) or >300 μg L^–1^ (HAL) in domestic well communities and reported 2011–2019 mean Mn values most proximal to point of entry in community water systems.

Within the total area served by DWCs in the Central Valley with predicted Mn in groundwater (15,123 km^2^), 671 km^2^ of the region has **≥**80% probability of groundwater extracted exceeding 50 μg L^–1^Mn and 180 km^2^ has **≥**80% probability of groundwater extracted exceeding 300 μg L^–1^ Mn (Figure 2). Since DWCs are less likely to be consuming treated drinking water, DWCs that overlap with areas of high probability of Mn in groundwater are likely accessing that water without treatment.^40,41^

The median domestic well depth varies depending on the hydrologic region. Within the northern Sacramento River basin, the median well depth in areas classified as a DWC is 41 m, within the central San Joaquin River basin the median is 61 m, and in the southern Tulare Lake region the median is 67 m (Table S4). We compared predicted Mn exceedances at a median depth of the associated hydrologic region and 17 m deeper in response to drought-related decline in water table.^16^ Our analyses demonstrated a significant difference between depths in the probability of Mn exceeding the SMCL for all regions (*p* < 0.001); however, the effect size was negligible (0.011; 95% CI: 0.027%, 0.03%). No significant difference was observed between depths for the Mn HAL in all regions (*p* = 0.252). The mean probability of exceeding the SMCL and HAL at the current depth of the associated hydrologic region is 17.2 and 7.9%, respectively, while the mean probability of exceeding the SMCL and HAL at 17 m deeper than the current depth is 17.5 and 7.9%, respectively (Table S6).

In comparison to DWC, CWSs are regularly monitored for primary contaminant MCL violations and are required to manage or treat groundwater until brought into compliance which may unintentionally treat for manganese. For example, in the Central Valley, where nitrate and arsenic contamination are major concerns, treatment methods such as ion exchange, reverse osmosis, and electrodialysis may also result in the removal of Mn.^42^ However, small CWSs often lack adequate treatment for primary contaminants and receive more MCL violations than larger systems in California.^13,26^ In some cases, domestic well users may live within a CWS boundary. To capture predicted Mn concentration for those users, we mapped the probability of shallow untreated groundwater concentrations exceeding threshold values in areas that are served by CWS (Figure S2).

### 3.3 Poverty in DWCs

**Figure 3:**
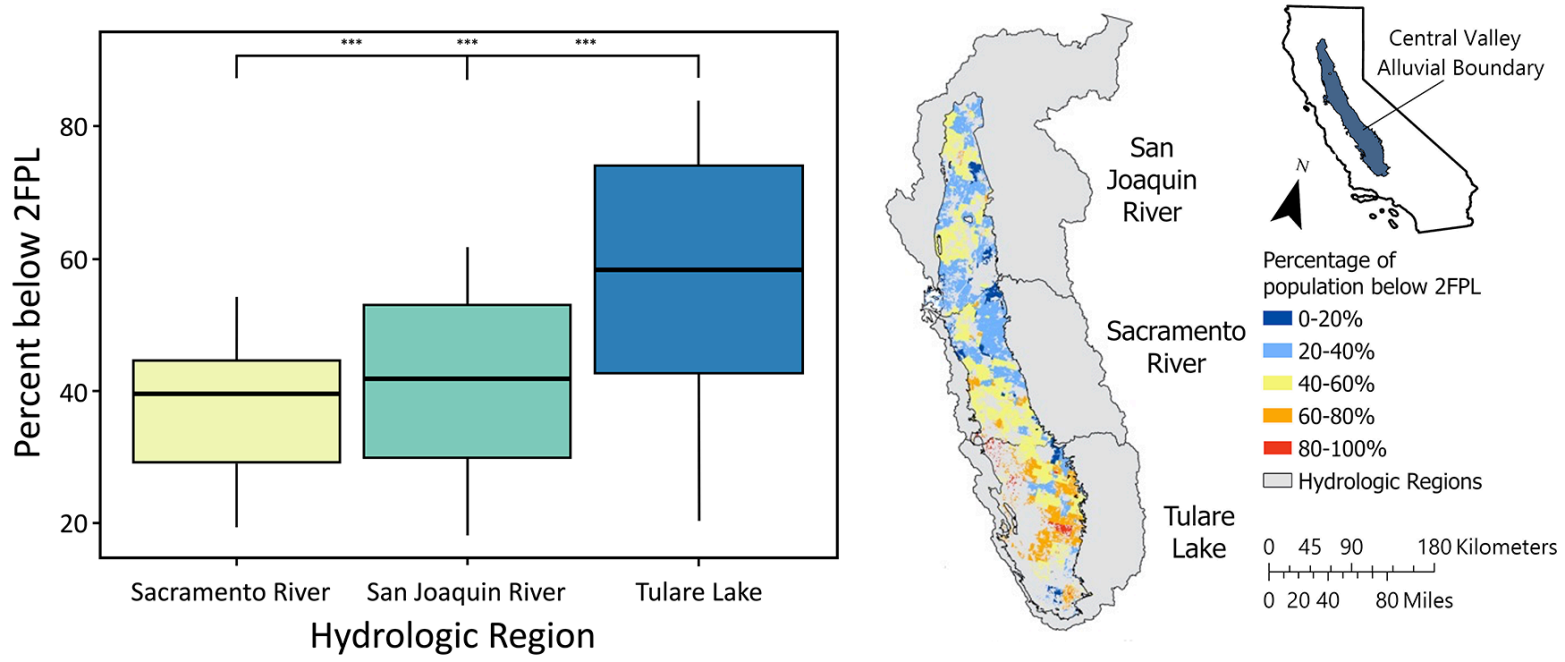
(A) Percentage of domestic well communities (DWC) below 2 times the federal poverty level (2FPL) in the Sacramento River, San Joaquin River, and Tulare Lake Hydrologic Regions. Boxes represent the 25th, 50th, and 75th percentiles of concentrations, and whiskers represent the 5th and 95th percentiles. Outliers were excluded. Bracket denotes a significant difference (*** indicates that *p* < 0.001) in the mean percentage of the population below 2FPL. DWCs in Sacramento River region: *n* = 5528, DWC in San Joaquin River region: *n* = 5210, DWCs in Tulare Lake Region: *n* = 5394. (B) Spatial distribution of DWCs within the Central Valley with population below 2FPL. Gray areas are the USGS Hydrologic Regions within the Central Valley.

We overlaid DWC boundaries with socioeconomic characteristics at the scale of census tracts and demonstrated that DWCs within the Central Valley region have higher occurrences of poverty than DWCs outside of this region (Figure 3). The mean poverty rate in DWCs within the Central Valley is 42.3% and is significantly higher (*p* < 0.001, Figure S7) than the DWCs outside of the Central Valley (32.4%; 95% CI: 9.6, 10.28; effect size = 0.72, Table S7). For reference, 31.3% of California residents have an annual household income that is below twice the federal poverty limit.^34^

The distribution of poverty throughout the Central Valley is highly heterogeneous, with a mean poverty rate of 56.6% in the Tulare Lake hydrologic region in Southern Central Valley (Figure 3). This is significantly higher (*p* < 0.001) than the San Joaquin River basin (41.4%; 95% CI: 14.6, 15.9; effect size = 0.89) and the northern Sacramento River basin (37.8%; 95% CI: 18.2, 19.2; effect size = 1.2, Tables S8 and S9). Community water system users may have access to municipal monitoring and treatment, but out-of-pocket costs for domestic well communities may be a considerable financial burden, particularly for those below the poverty line.

While poverty level is determined only by annual income, the label of disadvantaged community (DAC) is defined by CalEPA as communities overburdened by pollution, socioeconomic, and health challenges^36^ (SB 535). Our analysis estimates that approximately 48% of all domestic well users in the Central Valley live within DACs (Figure S10). Within that population, 88.8% of the DWC user population with a high (>80%) probability of extracting groundwater with Mn above the HAL live within a DAC (Table S11). Individuals within DAC communities may face further challenges in addressing water quality disparities including associated treatment costs or a larger pollution burden.

### 3.4 Possible Mn Mitigation Strategies

For communities that rely on domestic wells and CWS users lacking treatment, we have investigated three possible mitigation strategies for Mn overexposure: drilling of deeper wells, point-of-use treatment, or consolidation of DWCs into existing CWSs.

#### 3.4.1 Changing Well Depth in DWCs

Our results within this study suggest that drilling deeper wells is not an effective mitigation strategy for Mn concentration in groundwater used for drinking within the Central Valley region. A statistical significance (*p* < 0.001) was observed when comparing the probability of exceeding the SMCL in wells at the current median depth and 33 m deeper than the current median depth for all regions (*p* < 0.001) but was not statistically different when comparing the probability of exceeding the HAL in all regions (*p* = 0.225, Figure S3, Table S6). The mean probability of exceeding the SMCL at the current median depth is 17.2% while the mean probability of an exceedance 33 m deeper is 17.7% (Table S6). However, the effect size is negligible (0.02; 95% CI = 0.5%, 0.6%). Although we observe statistically significant results for the Sacramento River, Tulare Lake, and San Joaquin River regions, when we consider each hydrologic region individually, the effect size remains negligible across all Mn concentration thresholds (Table S6). Therefore, drilling deeper wells does not appear to protect against higher Mn concentrations in groundwater extracted for drinking and any costs associated with drilling deeper wells would have minimum possible benefit.

This contrasts with our original hypothesis and previous studies demonstrating more favorable geochemical conditions for Mn mobilization at shallow depths. Manganese mobility in subsurface environments is predominantly controlled by biotic and abiotic redox transformations that result in Mn immobilization through precipitation and adsorption reactions^1,43,44^ or mobilization via microbially driven reductive dissolution during anaerobic respiration.^45^ This kinetic constraint can result in high concentrations of Mn in shallow, oxic groundwaters^3,14,43,44^ and accumulation of Mn at relatively shallow aquifer depths over time.^1,41^ In addition, anoxia at deeper aquifer depths can lead to the accumulation of carbonate from anaerobic microbial respiration, which can immobilize Mn(II) through precipitation of Mn(II) carbonates.^46,47^

Despite prior work demonstrating higher Mn concentrations within shallow aquifer zones, we may not have observed this within our study since wells are drilled below the fluctuation redox zone within the shallow aquifer. Further, due to the absence of reported Mn concentrations in DWCs, we relied on predicted Mn values and therefore our results are impacted by model limitations. Wells are drilled to depths that ensure the screened portion is installed deeper than the variably saturated zone to ensure the water supply is not seasonally affected. In an analysis of groundwater Mn concentrations in wells throughout the United States, the highest concentrations were measured in observation wells, which are shallower than the domestic wells, and a smaller difference was observed between domestic and public supply wells.^2^ Within the Central Valley, studies investigating redox controls on chromium(VI) contamination of groundwater observed the highest rate of Mn oxide staining in the shallow aquifer zone between 10 and 20 m, which is shallower than the average domestic well in the region.^48,49^ Since the average well depths for DWCs were below depths where Mn accumulation was observed in the region, drilling deeper will not be beneficial. Further regional consideration of the depth to the shallow aquifer zone or area of Mn accumulation may be needed to determine if well depth could be protective in other areas.

#### 3.4.2 Point-of-Use Treatment

Several point-of-use (POU) treatment technologies can be used to treat Mn, although their accessibility can be limited by the cost and maintenance of the unit. POU treatments through chemical, biological, and physical means can result in the effective removal of Mn.^50,51^ Previous analysis of the efficacy of at-home water treatment methods on the removal of Mn in domestic wells in Nevada demonstrated a reduction in the median Mn concentration post-treatment.^52^ All methods require regular monitoring and maintenance to ensure the treatment continues to remove Mn. Annual water monitoring costs can range from US$100 to US$400, and annual treatment costs of water outside of compliance can range from US$70 to US$400 or even more depending on the choice of treatment method and installation cost (Table S13).

Many low-income communities within San Joaquin Valley pay 4–10% of household income for water-related expenses.^53,54^ When including expenses related to purchasing bottled water, up to 95% of households exceed the California Department of Public Health (CDPH) water minimum affordability threshold of 1.5% of the median household income.^53^ For individuals below 2FPL (US$25,760), monitoring and treatment for high Mn may account for 0.5–4% of an individual’s household income, excluding additional costs associated with purchased water or treating for additional contaminants. In our analysis, a majority of DWC users at risk of withdrawing groundwater with concentrations exceeding the HAL occur within a disadvantaged community (Table S11). Our results also show the Tulare Lake hydrologic region has a significantly higher percentage of residents below 2FPL than the other two hydrologic regions within Central Valley; any hotspot areas predicted to have high groundwater Mn in the Tulare region should be prioritized when designating Mn monitoring and treatment funds.

Legislative changes to primary contaminant regulation may have co-benefits upon secondary contaminant remediation. It has been reported that a decrease in arsenic concentrations and CWSs was correlated with the implementation of stricter regulation of As (Final Arsenic Rule which decreased the MCL from 50 to 10 μg L^–1^) most likely due to increased As treatment or mixing of groundwater in CWSs.^55^ Treatment of As within CWS may also treat for and remove Mn from groundwater within CWSs, but still does not address high Mn concentrations in DWCs that do not receive treatment.^42^ The Final Arsenic Rule legislation led to a direct change in concentrations in drinking water and demonstrates that stricter regulation of groundwater contamination is a pathway to limit exposure.^55^ Legislation of other contaminants, such as Mn, may also be used to limit exposure derived from CWSs, but not unregulated domestic wells. Further investigation is needed to target domestic well communities at high risk of Mn exposure through drinking water and in areas characterized by high rates of poverty to understand the burden of cost disparities and target areas for effective distribution of grants and funding.

#### 3.4.3 Consolidation of DWCs

Consolidation of domestic well communities and small state water systems represents an additional option to decrease high Mn as well as other contaminants in drinking water. In our analysis, DWCs had extremely low monitoring data availability (Table S2). Consolidation onto an existing CWS may result in treated water and better monitoring/reporting of water quality parameters. In an analysis of disadvantaged unincorporated communities in California's Central Valley relying on domestic wells or small water systems, 66% were within 4.8 km of an existing system where consolidation is considered feasible.^18^ Though the state cannot force consolidation for domestic well users, financial incentivization has led to the successful subsumption of domestic wells and small systems by the SWRCB for communities in Tulare and Kings County and successfully resolved arsenic MCL violations and well failure issues.^56^

Consolidation is a resource-intensive process and the allocation of funds to support consolidation can be used to counter a community’s reluctance to consolidate. The Safe and Affordable Drinking Water Fund (SB 200) was passed in 2019 and allocates $130 million per year to improve water infrastructure in disadvantaged communities, including consolidation projects.^57^ In addition, consolidation of smaller systems into larger systems may help bring down the cost of treatment due to improved economies of scale. Since larger systems serve more users, they may distribute the fixed costs over many more users thereby driving down average costs whereas similar capital cost investments in smaller systems may significantly increase average costs.^58^ Applying for consolidation funding and construction is a multiyear project and interim treatment or alternative water source is needed for communities currently experiencing drinking water quality violations.

### 3.5 Limitations

#### 3.5.1 Spatial and Temporal Limitations of Mn Groundwater Model

The probability model used in this paper considers Mn concentrations predominantly from shallow groundwater monitoring wells and relatively deeper public supply wells which do not necessarily reflect the chemistry of finished water that is delivered to users dependent on shallow, private wells.^27^ Further, shifts in subsurface geochemical conditions caused by environmental and human activity (e.g., seasonal variations and increased groundwater pumping possibly exacerbated by increasing drought frequencies) may influence contaminant release and were not considered here when assessing the probability of groundwater concentrations exceeding threshold values.^1^

Further consideration of the heterogeneity of well depth within DWCs is important for understanding redox-sensitive contaminants release into drinking water when applying depth-resolved contaminant models.

#### 3.5.2 Data Availability Limitations

A major limitation to better understanding groundwater Mn concentrations is the lack of publicly available data on Mn occurrence and concentration in groundwater serving domestic wells collected and managed by federal and state agencies. The Groundwater Ambient Monitoring Assessment (GAMA) program was established by the California SWRCB in 2000 to monitor and assess groundwater quality in areas that have a high reliance on groundwater resources. Since its inception, the program has monitored over 2,300 domestic wells for water quality throughout the state; however, only ∼5% of the wells were within the Central Valley and reported both well depth and Mn concentration^58^ (Table 2). In our analysis, 110 domestic wells reported depth-resolved Mn concentration data between 2000 and 2019 out of 72,800 domestic wells within the region (Table S4). The water quality data for only 0.15% of domestic wells will considerably underestimate populations accessing water exceeding acceptable contaminant concentrations. Collecting current and accurate water quality information from active wells is critical to both confirming large-scale predictive models and building more models to address other drinking water contaminants.

Another limitation is missing Mn data from CWS collected by state regulatory agencies. Currently, each CWS must monitor secondary contaminants in its groundwater sources on a 3-year basis or its surface water sources annually (22 CCR § 64449). Yet despite this regulatory monitoring framework, 44.1% of the CWS population in the Central Valley has no reported Mn concentration at point of entry in SDWIS between 2011 and 2019 and we are unable to assess water quality within these CWS (Table 2). Therefore, the estimated population accessing water with high Mn is likely an underestimate. To account for discrepancies in reporting frequency between CWSs and ensure each well point was weighted equally regardless of frequency, a 9-year, time-weighted average of the available data was used to estimate Mn concentration. However, this fails to capture variability in groundwater Mn concentration driven by changes in seasons, water use, or precipitation.

Another important data gap pertains to a paucity of data on the location of disadvantaged unincorporated communities (DUCs) which rely on informal or small water systems. These communities may live within CWS boundaries but lack infrastructure to access formal CWSs and associated monitoring/treatment infrastructure.^18,56^ Previous studies have assumed the entire population within CWS boundaries are supplied by CWSs,^25,26^ but no study has investigated the infrastructure available within DUCs and the population served by such infrastructure throughout the entire state. These communities, therefore, warrant additional attention in future studies addressing water quality disparities driven by resource or infrastructure disparities.

## 4 Future Implications

This current work is an exploratory analysis but demonstrates a deep need for better quality and publicly available data to further investigate and understand Mn contamination for domestic well communities and community water systems. California has made clear its commitment to providing safe, clean, affordable, and accessible drinking water to those who live within the state, but more monitoring of domestic drinking water wells and support of domestic well users need to be prioritized not just for Mn, but all contaminants.

Many nonprofits and community advocacy groups are already asking for increased focus on domestic wells and their users. These groups already have trusted relationships within the local community and access to well locations previously inaccessible to government agencies. When building large-scale monitoring or mitigation projects, attention to what is already being done by these groups will be extremely useful. In addition, increased funding and collaborations with these groups will also increase access to users’ ability to mitigate water quality issues at a more individual level.

## Supporting Information Available

List of publicly available data used (Table S1); count of available Mn concentration data during study period (Tables S2 and S3); distribution of predicted Mn exceedances in DWC and CWS (Figure S1); Summary of private well depths in central valley hydrologic regions (Table S4); reported Mn water quality in domestic wells (Table S5); summary of statistical results (Tables S6–S10); probability of Mn exceedance for DWC users within CWS boundary (Figure S2); percentage of domestic well communities (Figure S3); DWC poverty rates within and outside of the Central Valley’s DWCs (Figure S4); summary of DWCs within or outside disadvantaged communities (Figure S5 and Table S11); additional population estimates for domestic well communities (Table S12); and point-of-use treatments for Mn (Table S13) (PDF)
