# Supplementary material for: Disparities in Drinking Water Manganese Concentrations in Domestic Wells and Community Water Systems in the Central Valley, CA, USA
Source: Environ Sci Technol. 2023 Jan 25;57(5):1987–96. doi: 10.1021/acs.est.2c08548 (PMC9910038; doi:10.1021/acs.est.2c08548)
Supplement: Supplementary file 1 — es2c08548_si_001.pdf [file es2c08548_si_001.pdf]

Supporting Information

Disparities in drinking water manganese concentrations in domestic wells and community water systems in the Central Valley, CA, USA

Miranda L. Aiken[1,2], Clare Pace[3], Maithili Ramachandran[4], Kurt Schwabe[4], Hoori Ajami[5], Bruce Link[4], Samantha C. Ying[1,5,6*]

[1]Environmental Toxicology Graduate Program, University of California, Riverside, CA 92521
[2]Schmid College of Science and Technology, Chapman University, California, 92866, United States of America
[3]Environmental Science, Policy, and Management, University of California, Berkeley, CA, 94704
[4]School of Public Policy, University of California, Riverside, CA 92521
[5]Environmental Sciences Department, University of California, Riverside, CA 92521
[6]Health Disparities Research Center, School of Medicine, University of California, Riverside, CA 92521

*Corresponding author: Samantha Ying; samying@ucr.edu

Supporting Information Content (16 pages, 13 tables, 4 figures)

Section I: List of publicly available data used ……………………………………...………..S2
Table S1
Section II: Count of available Mn concentration data during study period ………......…..……S3
Table S2
Table S3
Section III: Distribution of predicted Mn exceedances in DWC and CWS ………………...…S4
Figure S1
Section IV: Summary of Private Well Depths in Central Valley Hydrologic Regions S5
Table S4
Section V: Reported Mn Water Quality in Domestic Wells …………………..………..…..…..S6
Table S5
Section VI: Summary of Statistical Results …………………………………..…..…………..S7
Table S6
Table S7
Table S8
Table S9
Table S10
Section VII: Probability of Mn Exceedance for DWC users within CWS boundary ...……..…S11
Figure S2
Section VIII: DWC Poverty Rates within and outside of the Central Valley's DWCs ..…..…..S12
Figure S4
Section IX: Summary of DWCs within or outside disadvantaged communities ………..……...…S13
Figure S5
Table S11
Section X: Additional Population Estimates for Domestic Well Communities.........................S14

Table S12
Section XI: Point of use treatments for Mn …………………………………..…...………..S15
Table S13
Section XII: References ……………………………………………………...……...…S16

Section I: List of publicly available data used

Table S1. Publicly available data sources.

**Description**	**Data Source**
Likely DWC Boundaries	https://drinkingwatertool.communitywatercenter.org/data/
CWS Boundary	https://drinkingwatertool.communitywatercenter.org/data/
Groundwater Mn Predictive Model (33 m, 67 m, 100 m)	https://www.sciencebase.gov/catalog/item/57f433c3e4b0bc0bec033fc9
Reported Mn Concentrations at Point of Entry	https://www.waterboards.ca.gov/drinking_water/certlic/drinkingwater/EDTlibrary.html
US Census population	https://drinkingwatertool.communitywatercenter.org/data/
CWS user population	https://data.ca.gov/dataset/drinking-water-public-water-system-information
Percentage below 2FPL (OEHHA)	https://oehha.ca.gov/calenviroscreen/indicator/poverty
Disadvantaged Community Designation	https://data.ca.gov/dataset/sb-535-disadvantaged-communities-2017

Section II: Count of available Mn concentration data during study period

Table S2: Count of available Mn data from Safe Drinking Water Information System (California State Water Resources Control Board, 2021) between 2011-2019. CWS = community water system.

Data Processing Step	Total	Small[A] CWS	Medium[B] CWS	Large[C] CWS
Count of reported Mn for active community water systems statewide	138,072	-	-	-
Count of reported Mn for active community water systems in Central Valley [D]	28,852	3,916	9,977	14,959
Count of reported Mn for active community water systems in Central Valley at point most proximal to point-of-entry [D]	13,113	2,323	4,736	6,054
Total active CWS in Central Valley with reported Mn	639	398	188	53
Total active CWS in Central Valley with reported Mn at point-of-entry	373	245	87	41

[A]Small CWS = 15-199 service connections
[B]Medium CWS = 200-9,999 service connections
[C]Large CWS > 10,000 service connections.
[D]All CWS with 0 reported population and <15 service connections were removed from analysis (n=400 reported Mn concentrations within the Central Valley)

Table S3. Summary of mean Mn concentrations (µg L^{-1}) between 2011-2019 for small, medium, and large CWS.

	Small CWS	Medium CWS	Large CWS
Median (IQR)[A]	14.2 (16.2)	14.3 (2.7)	14.3 (0.9)
Mean (Std. Dev.)	52.5 (98.7)	26.7 (46.9)	18.7 (27.8)
95th Percentile	224.4	65.2	30.5

[A]Interquartile range

Section III: Distribution of predicted Mn exceedances in DWC and CWS

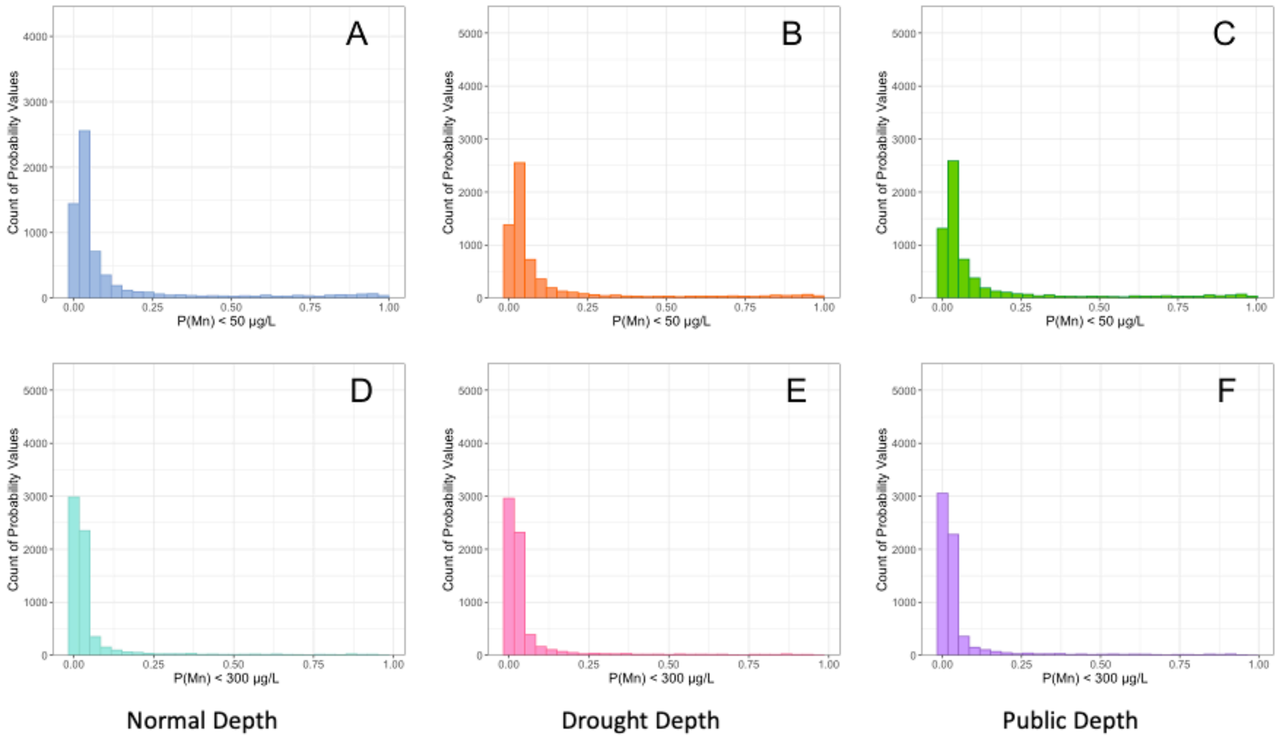

Figure S1. Distributions of the probability of groundwater Mn exceeding secondary contaminant level and health advisory limits in DWC. Predicted Mn exceedance values were retained at the median private well depths for each hydrologic region in the Central Valley (Sacramento River – 33 m, San Joaquin River – 50 m, Tulare lake – 66 m). Probability of exceeding the 50 µg L^{-1} at the current median well depth (A), drought depth (17 m deeper, B), and public well depth (33 m deeper, C). Probability of exceeding the 300 µg L^{-1} at the current median well depth (D), drought depth (17 m deeper, E), and public well depth (33 m deeper, F)

Section IV: Summary of Private Well Depths in Central Valley Hydrologic Regions

To determine the depth best representative of private wells, well depth grids from Voss et al. (2018) and Kauffman et al. (2021) were assigned to each hydrologic region.

		Entire Central Valley Alluvial Boundary			Only Domestic Well Communities[A]		
		Sacramento River	San Joaquin River	Tulare Lake	Sacramento River	San Joaquin River	Tulare Lake
**Voss et al. 2018**	**Mean (Std. Dev.)**	131.11 (54.45)	172.03 (58.78)	253.00 (126.34)	129 (58.17)	167 (47.95)	208 (102.8)
	**Median**	121.78	167.8	222.64	118	167	192
	**Maximum**	490.89	709.02	826.93	490.89	679.04	826.93
	**95th Percentile**	233.52	253.31	505.72	237.49	235.95	403.21
**Kauffman et al. 2021**	**Mean (Std. Dev.)**	137.34 (60.71)	182.72 (70.23)	258.04 (128.70)	138 (62.51)	182 (66.76)	228 (123.35)
	**Median (IQR)**	124.72	181.78	245.69	124	182	200
	**Maximum**	440	530.56	904	434.67	500.56	904
	**95th Percentile**	254.5	297.22	500	266.32	293.25	466.97

[A]Domestic well community delineation from Pace et al. (2020)

Table S4. Summary of private well depth within the entire Central Valley alluvial boundary and only within the domestic well communities. All values reported in feet.

Section V: Reported Mn Water Quality in Domestic Wells

Table S5. Reported Mn ($\mu g\ L^{-1}$) concentrations in domestic wells (1 to 4 service connections) between 2001-2019. All water quality estimates from wells between 15 - 61 m were included in estimates for ambient Mn water quality.

	Domestic Wells[A]
**Number of domestic wells in the Central Valley[B]**	72,846
**Total reported Mn values**	125
**Total wells with reported Mn values**	110
**Number of wells with repeated observations**	12
**Median (IQR)**	0.58 (17.5)
**Mean (Std. Dev.)**	61.2 (171.38)
**Max**	1130
**95th percentile**	365.8

[A]Water quality data was collected from United States Geological Survey (USGS), National Water Information System (NWIS), and California State Water Board Groundwater Ambient Monitoring Assessment (GAMA) database (California State Water Resources Control Board, 2020) and taken from: https://ca.water.usgs.gov/gama/water-quality-results/. All data with Mn concentration and depth data were retained.

[B]Count of wells within each DWC from Pace et al., (2020)

Section VI: Summary of Statistical Results

Although the data was non-normally distributed, non-parametric tests are less useful in studies with large sample sizes. In Fagerland (2012), simulations of parametric and non-parametric tests on gamma and log-normal distributed data were run. They found that the larger the sample size, the higher the rejection rate for non-parametric tests, whereas the rejection rate for parametric remained similar. Therefore, despite the non-normal distribution of our data, we used a parametric t-test to determine if there was a difference in the mean of probability of groundwater Mn exceeding SMCL and HAL. When comparing reported values at two depths, a paired t-test was used to determine the difference in mean within the sample. A paired Cohen's d was also determined to describe the magnitude of the difference in means (Sullivan and Feinn, 2012). All statistical analyses were performed using R (version 4.0.5).

Table S6: Summary of paired t-test between the median depth of domestic wells in each Central Valley Hydrologic Region (Sacramento River – 33 m, San Joaquin River – 50 m, Tulare Lake – 66 m) and the predicted depth if wells were drilled deeper during times of drought (17 m deeper) and to the depth of public wells (33 m deeper) as a protective measure against contamination.

		Mn conc. > 50 ug/L				Mn conc. > 300 ug/L			
		**All Regions**	**Sacramento River**	**Tulare Lake**	**San Joaquin River**	**All Regions**	**Sacramento River**	**Tulare Lake**	**San Joaquin River**
		n = 12,349	n = 4,515	n = 3,616	n = 4,218	n = 12,349	n = 4,515	n = 3,616	n = 4,218
		Mean				Mean			
**Normal Depth**		0.172	0.208	0.117	0.180	0.079	0.080	0.047	0.107
**Drought (17 m deeper)**		0.175	0.212	0.117	0.184	0.079	0.080	0.046	0.107
**Public well depth (33 m deeper)**		0.177	0.208	0.121	0.186	0.079	0.078	0.046	0.109
**Difference in means normal vs. predicted drought**		0.003	0.004	0.001	0.003	0.000	0.000	-0.001	0.000
**Difference in means normal vs. public well depth**		0.005	0.000	0.004	0.005	0.000	-0.002	-0.001	0.002
**17 m deeper**	t-statistic	31.229	23.302	6.056	20.623	-1.146	-1.000	-3.838	1.450
	p-value (95 % CI)	0.000 (0.0027 to 0.00030)	0.000 (0.0039 to 0.0046)	0.000 (0.0003 to 0.0006)	0.000 (0.003 to 0.004)	0.252 (-0.0005 to 0.0001)	0.317 (-0.0010 to 0.0003)	0.000 (-0.0008 to -0.0002)	0.146 (-0.0001 to 0.0007)
	Effect size (95% CI)	0.011 (-0.02 to 0.05) negligible	0.014 (-0.03 to 0.06) negligible	0.002 (-0.04 to 0.05) negligible	0.013 (-0.03 to 0.05) negligible	0.001 (-0.03 to 0.04) negligible	-0.002 (-0.04 to 0.04) negligible	-0.005 (-0.05 to 0.04) negligible	0.001 (-0.04 to 0.04) negligible
**33 m deeper**	t-statistic	49.290	31.287	36.185	26.440	-1.214	-5.012	-4.497	5.350
	p-value	0.000 (0.005 to 0.006)	0.000 (0.006 to 0.007)	0.000 (0.0036 to 0.004)	0.000 (0.0051 to 0.0059)	0.225 (-0.0006 to 0.0001)	0.000 (-0.0025 to -0.0011)	0.000 (-0.0008 to -0.0003)	0.000 (0.0011 to 0.0024)
	Effect size (95% CI)	0.02 (-0.02 to 0.05) negligible	0.022 (-0.03 to 0.06) negligible	0.018 (-0.03 to 0.06) negligible	0.020 (-0.03 to 0.06) negligible	-0.001 (-0.04 to 0.03) negligible	-0.013 (-0.05 to 0.03) negligible	-0.005 (-0.05 to 0.04) negligible	0.008 (-0.03 to 0.06) negligible

To determine the difference in the percentage of the population in DWC below 2FPL within the Central Valley and outside of the Central Valley, we used a Welch t-test due to the variation within the sample size. The effect size was determined using Cohen's d.

Table S7: Summary of Welch t-test between percentage of DWC communities within and outside of the Central Valley that are below 2FPL.

		**Percentage below 2FPL**
Groups	**DWC Count**	**Mean**
DWC in the Central Valley	n= 6,953 (26.2%)	42.3
DWC outside the Central Valley	n=19,528 (73.8%)	32.4
	**Difference**	9.9
	**t-statistic**	-47.51
	**p-value (95%CI)**	0.000 (9.46 to 10.28)
	**effect size (95% CI)**	0.72 (0.70 to 0.75) medium

To determine the difference between the population in DWCs below 2FPL in the three hydrological regions in the Central Valley, a Kruskal-Wallis and Dunn test was used to test the difference in the medians. The effect size was determined using Cohen's d.

Table S8. Summary of Dunn test between the poverty rates in the Central Valley hydrologic regions. DWCs in Sacramento River region: n=5,528, DWC in San Joaquin River region: n=5,210, DWCs in Tulare Lake Region: n=5,394.

**Hydrologic Region**	**P-Value**	**Effect Size**
**Tulare Lake and Sacramento River**	0.000 (18.2 to 19.2)	1.2 (large)
**Sacramento River and San Joaquin River**	0.000 (3.1 to 4.1)	0.28 (small)
**San Joaquin River and Tulare Lake**	0.000 (14.6 to 15.9)	0.89 (large)

Table S9. Summary of poverty rates in the Central Valley hydrologic regions. DWCs in Sacramento River region: n=5,528, DWC in San Joaquin River region: n=5,210, DWCs in Tulare Lake Region: n=5,394.

Hydrologic Region	Median	Minimum	Maximum	Standard Deviation
**Sacramento River**	39.6	0	71.6	11.1
**San Joaquin River**	41.4	9.9	87	14.4
**Tulare Lake**	56.6	0	100	19.3

To determine the difference in the percentage of the population in DWC below 2FPL within the Central Valley and outside of the Central Valley, we used a Welch t-test due to the variation within the sample size. The effect size was determined using Cohen's d.

Table S10: Summary of Welch t-test between the probability of the Mn concentration exceeding threshold values within and outside of Central Valley disadvantaged communities.

		Mn conc. > 50 ug/L	Mn conc. > 300 ug/L
**Well depth**	**DWC s**	**Mean**	**Mean**
**DWC in DAC**	n=2998	0.167	0.089
**DWC outside DAC**	n=3421	0.167	0.067
	Difference	0.000	0.006
	**t-statistic**	2.41	6.48
	**p-value**	0.016<br>(0.002 to 0.023)	0.000<br>(0.015 to 0.029)
	**effect size (95% CI)**	0.06<br>(0.01 to 0.11)<br>negligible	0.17<br>(0.12 to 0.21)<br>negligible

Section VII: Probability of Mn Exceedance for DWC users within CWS boundary

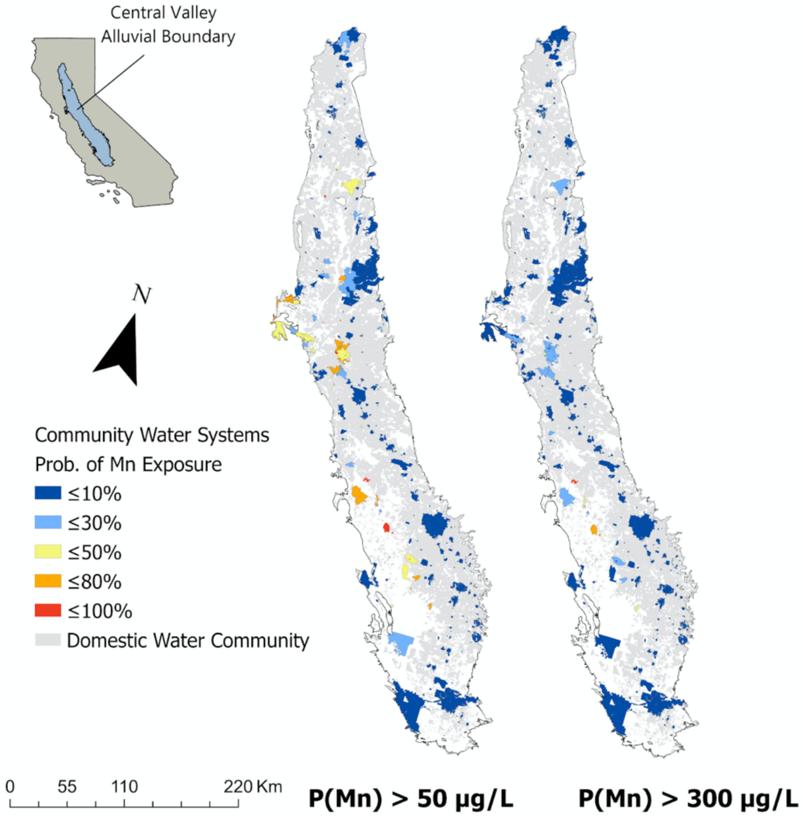

Figure S2. Probability of Mn in groundwater exceeding 50 µg L^{-1} or 300 µg L^{-1} for shallow (33 m) domestic well users residing within CWS boundaries. Mn predictive model is from Rosecrans et al., (2017) and CWS boundaries are from Pace et al., (2020).

Section VII: DWC Poverty Rates within and outside of the Central Valley's DWCs

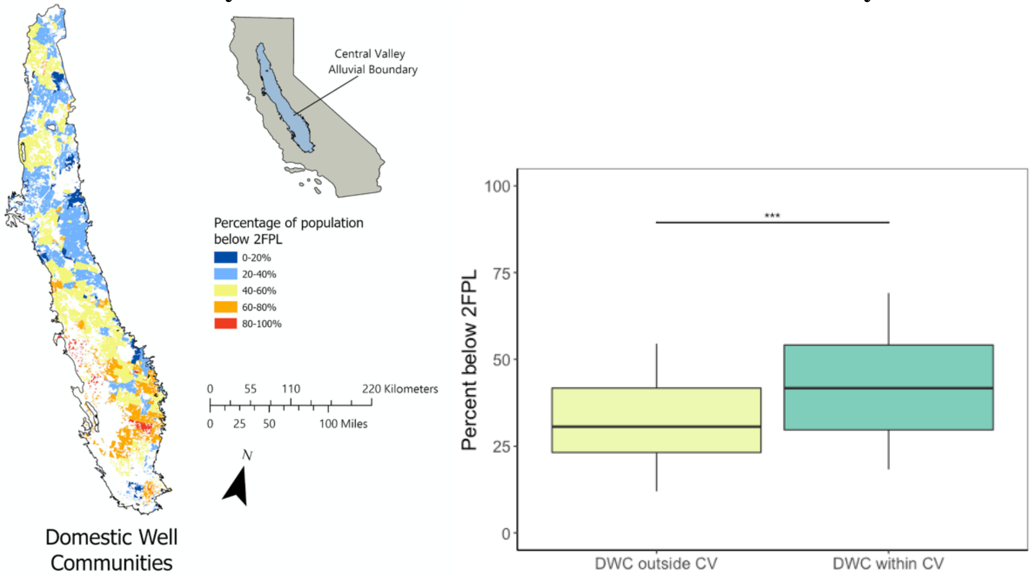

Figure S3. (A) Percentage of domestic well communities (DWC) below 2 times the federal poverty level (2FPL) outside of the Central Valley (CV) and within the CV. Boxes represent the 25th, 50th, and 75th percentile of concentrations and whiskers represent 5th and 95th percentiles. Outliers were excluded. Bracket shows denotes that a significant difference (*** indicates that $p < 0.001$) in the mean percentage of the population below 2FPL. DWC outside of CV: n=6,953, DWC within CV: n=19,528. (B) Spatial distribution of DWCs within the Central Valley with population below 2FPL.

Section IX: Summary of DWCs within or outside disadvantaged communities

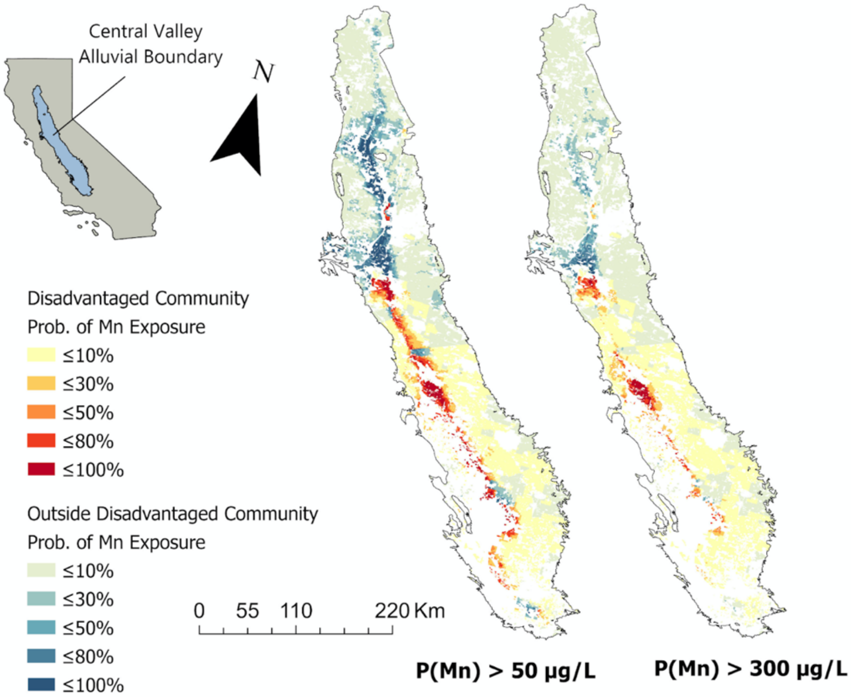

Figure S4. Probability of groundwater Mn exceeding 50 µg L^{-1} or 300 µg L^{-1} within disadvantaged communities as designated by CalEPA and defined by the communities that are most likely to suffer from economic, health, and environmental burdens (SB 535). Data sourced from **https://data.ca.gov/dataset/sb-535-disadvantaged-communities-2017** and last updated in 2017.

Table S11. Domestic well community population within and outside of disadvantaged communities.

	**Within DAC**	**Outside DAC**
**Total population**	268,706	285,730
**Total wells**	24,365	43,621
**Population (%) >80% 50 µg/L Mn**	4,066 (1.5)	18,603 (6.5)
**Population (%) <80% 50 µg/L Mn**	264,640 (98.5)	267,127 (93.5)
**Population (%) >80% 300 µg/L Mn**	2,079 (0.8)	263 (0.1)
**Population (%) <80% 300 µg/L Mn**	266,627 (99.2)	285,467 (99.9)

Section X: Additional Population Estimates for Domestic Well Communities.

In order to compare population estimates for DWCs from different methods, raster data from Johnson et al. (2020) was attributed to each DWC and population with high (>80%) likelihood of exceeding Mn threshold concentrations in extracted groundwater were estimated for each hydrologic basin within the Central Valley. Population for each DWC was estimated via aerial apportionment of raster cells predicting domestic well population from 1990 U.S. Census, when water source was reported, to population data collected from the 2010 U.S. Census (Johnson et al. 2020).

	**All DWC**	**Sacramento River DWC**	**San Joaquin River DWC**	**Tulare Lake DWC**
Total population with predictive model values	311,981	86,745 (27.8)	132,321 (42.7)	92,915 (28.8)
Population (%) >80% probability of exceeding 50 μg/L Mn	6,505 (2.1)	3,309 (3.8)	2,541 (1.9)	555 (0.6)
Population (%) <80% probability of exceeding 50 μg/L Mn	318,486 (97.9)	83,436 (96.2)	129,780 (98.1)	92,360 (99.4)
Population (%) >80% probability of exceeding 300 μg/L Mn	1,705 (0.5)	83 (0.1)	1593 (1.2)	29 (0.03)
Population (%) <80% probability of exceeding 300 μg/L Mn	10,276 (99.5)	86,662 (99.9)	130,728 (99.9)	93,886 (99.97)

Table S12. Population estimates from Johnson et al. (2019) for DWCs with high (>80%) probability of exceeding threshold values in extracted groundwater.

Section XI: Possible point of use treatments for Mn in drinking water

Table S13: Summary of possible point-of-use treatments for Mn in drinking water and costs associated.

Method	Description	Disadvantages	References for Method	Annual cost for point of use treatment
Oxidation, precipitation, and removal	Chemical oxidation and precipitation of dissolved Mn (II) and filtration of Mn solids; common oxidants include oxygen, chlorine dioxide, ozone, or permanganate	Generates sludge which must be properly disposed of; strong oxidant or high pH required to ensure effective removal; competition with other oxidant demands (e.g. iron or organic carbon); water must be monitored to ensure effective oxidant dosing	(Wong, 1984; Knocke et al., 1988)	Air injecting filter (0.3 ppm removal): \$220-300 per year<br>Chlorination and filtration (2 ppm removal): \$100-120 per year
Sorption	Sorption of dissolved Mn on solid surface (often metal or Mn oxide, such as "greensand" filter); can be combined with catalytic oxidation by free chlorine to continuously regenerate Mn oxide surface	Once surface is saturated, breakthrough occurs and must be monitored; filter must be regenerated or replaced once breakthrough occurs	(Knocke et al., 1987, 1988; Wang et al., 2021)	Greensands Filter (5 ppm removal): \$300-400 per year<br>Iron or Mn filter media (0.3 ppm removal): \$50-80 per year
Ion exchange	Cation exchange resins (strong-acid exchangers or weak-acid exchangers) bind divalent ions present in water (e.g. Ca^{2+}, Zn^{2+}, Fe^{2+}, Mg^{2+} , and Mn^{2+})	Other divalent cations present in the water may interfere with binding or leach sorbed Mn(II), often a problem in water with high hardness; exchange media must be replaced; pour over filter only effective for low flow volumes	(Carrière et al., 2011)	Water softener (0.5 ppm): \$70-200 per year<br>Pour over filter (0.5 ppm): \$70 per year
Membrane filtration	Water is passed through a semipermeable membrane (e.g. microfiltration, nanofiltration, reverse osmosis, ultrafiltration)	Membrane must be maintained or replaced often; slow filtration depending on membrane permeability; potential membrane fouling	(Teng et al., 2001; Choo et al., 2005)	Reverse osmosis (ppm): \$100-200 per year
Biological removal	Water is passed through media with biofilm containing microbes able to directly oxidize Mn(II), adsorb it extracellularly, or catalyze Mn(II) oxidation through biopolymers	Proper nutrient solution must be maintained to sustain the biofilm; varying acclimation time required; continuous monitoring required	(Gouzinis et al., 1998; Hoyland et al., 2014; Breda et al., 2017; Ramsay et al., 2018)	Not commonly used as point of use treatment
Infrastructure management	Modifications to wells to prevent high exposure to Mn such as blending water, installing deeper wells, or monitoring of private wells to take them offline when in exceedance	Requires monitoring of all private and public wells; may not be feasible for private wells where cost is carried by owner	(Tobiason et al., 2016)	Drilling deeper well (90.06 m): \$30-65/ft, \$9,000 to 17,500 total<br>Joining municipal water system: \$1,500 to 20,000 total depending on location<br>Water monitoring cost: \$100-400 per sample

Section XII: Appendix References

Breda I. L., Ramsay L. and Roslev P. (2017) Manganese oxidation and bacterial diversity on different filter media coatings during the start-up of drinking water biofilters. *Journal of Water Supply: Research and Technology-Aqua* **66**, 641–650.

California State Water Resource Control Board (2021) EDT Library and Water Quality Analyses Data and Download Page. https://www.waterboards.ca.gov/drinking_water/certlic/drinkingwater/EDTlibrary.html (accessed January 3rd, 2022)

California State Water Resource Control Board (2020) Groundwater Ambient Monitoring and Assessment (GAMA).37. https://ca.water.usgs.gov/gama/water-quality-results/. (accessed January 3rd, 2022)

Carrière A., Brouillon M., Sauvé S., Bouchard M. F. and Barbeau B. (2011) Performance of point-of-use devices to remove manganese from drinking water. *Journal of Environmental Science and Health, Part A* **46**, 601–607.

Choo K.-H., Lee H. and Choi S.-J. (2005) Iron and manganese removal and membrane fouling during UF in conjunction with prechlorination for drinking water treatment. *Journal of Membrane Science* **267**, 18–26.

Fagerland M. W. (2012) t-tests, non-parametric tests, and large studies—a paradox of statistical practice? *BMC Medical Research Methodology* **12**, 78.

Gouzinis A., Kosmidis N., Vayenas D. V. and Lyberatos G. (1998) Removal of Mn and simultaneous removal of NH3, Fe and Mn from potable water using a trickling filter. *Water Research* **32**, 2442–2450.

Hoyland V. W., Knocke W. R., Falkinham J. O., Pruden A. and Singh G. (2014) Effect of drinking water treatment process parameters on biological removal of manganese from surface water. *Water Res* **66**, 31–39.

Knocke W. R., Hoehn R. C. and Sinsabaugh R. L. (1987) Using Alternative Oxidants to Remove Dissolved Manganese From Waters Laden With Organics. *Journal AWWA* **79**, 75–79.

Knocke W. R., Ramon J. R. and Thompson C. P. (1988) Soluble Manganese Removal on Oxide-Coated Filter Media. *Journal (American Water Works Association)* **80**, 65–70.

Pace C., Balazs C., Cushing L. and Morello-Frosch R. (2020) Locating Domestic Well Communities in California: A Methodological Overview. https://drinkingwatertool.communitywatercenter.org/wp-content/uploads/2020/01/White-paper_FINAL_2020.1.14.pdf

Ramsay L., Breda I. L. and Søborg D. A. (2018) Comprehensive analysis of the start-up period of a full-scale drinking water biofilter provides guidance for optimization. *Drinking Water Engineering and Science* **11**, 87–100.

Rosecrans C. Z., Nolan B. T. and Gronberg J. M. (2017) Prediction and visualization of redox conditions in the groundwater of Central Valley, California. *Journal of Hydrology* **546**, 341–356.

Sullivan G. M. and Feinn R. (2012) Using Effect Size—or Why the P Value Is Not Enough. *J Grad Med Educ* **4**, 279–282.

Teng Z., Yuan Huang J., Fujita K. and Takizawa S. (2001) Manganese removal by hollow fiber micro-filter. Membrane separation for drinking water. *Desalination* **139**, 411–418.

Tobiason J. E., Bazilio A., Goodwill J., Mai X. and Nguyen C. (2016) Manganese Removal from Drinking Water Sources. *Curr Pollution Rep* **2**, 168–177.

Wang P., Wang H., Zhang Y., Yi J., Chen M., Jiang H., Yan J., Liu H. and Ma J. (2021) Accelerated catalytic oxidation of dissolved manganese(II) by chlorine in the presence of in situ-growing 3D manganese(III)/(IV) oxide nanosheet assembly in zeolite filter. *Water Res* **201**, 117223.

Wong J. M. (1984) Chlorination-Filtration for Iron and Manganese Removal. *Journal AWWA* **76**, 76–79.
